# CXCL16 positively correlated with M2-macrophage infiltration, enhanced angiogenesis, and poor prognosis in thyroid cancer

**DOI:** 10.1038/s41598-019-49613-z

**Published:** 2019-09-16

**Authors:** Min Joo Kim, Hyun Jin Sun, Young Shin Song, Seong-Keun Yoo, Young A Kim, Jeong-Sun Seo, Young Joo Park, Sun Wook Cho

**Affiliations:** 1 Department of Internal Medicine, Seoul National University Hospital, 101, Daehak-ro, Jongno-gu, Seoul, 03080 Republic of Korea; 2 Department of Internal Medicine, CHA Bundang Medical Center, CHA University, 59, Yatap-ro, Bundang-gu, Seongnam, Republic of Korea; 3 Gong Wu Genomic Medicine Institute, Seoul National University Bundang Hospital, Dolma-ro 172, Seongnam, 13605 Republic of Korea; 4 Department of Pathology, Boramae Medical Center, 20, Boramae-ro 5-gil, Dongjak-gu, Seoul, 07061 Republic of Korea; 5 Department of Internal Medicine, Seoul National University College of Medicine, 101, Daehak-ro, Jongno-gu, Seoul, 03080 Republic of Korea

**Keywords:** Thyroid cancer, Cancer microenvironment, Thyroid diseases

## Abstract

Although various chemokines have pro-tumorigenic actions in cancers, the effects of CXCL16 remain controversial. The aim of this study was to investigate the molecular characteristics of CXCL16-expressing papillary thyroid cancers (PTCs). CXCL16 expressions were significantly higher in PTCs than benign or normal thyroid tissues. In the TCGA dataset for PTCs, a higher *CXCL16* expression was associated with M2 macrophage- and angiogenesis-related genes and poor prognostic factors including a higher TNM staging and the *BRAF*^V600E^ mutation. PTCs with a higher expression of 3-gene panel including *CXCL16*, *AHNAK2*, and *THBS2* showed poor recurrence-free survivals than that of the lower expression group. Next, shCXCL16 was introduced into BHP10-3SCp cells to deplete the endogenous CXCL16, and then, the cells were subcutaneously injected to athymic mice. Tumors from the BHP10-3SCp^shCXCL16^ exhibited a delayed tumor growth with decreased numbers of ERG^+^ endothelial cells and F4/80^+^ macrophages than those from the BHP10-3SCp^control^. CXCL16-related genes including *AHNAK2* and *THBS2* were downregulated in the tumors from the BHP10-3SCp^shCXCL16^ compared with that from the BHP10-3SCp^control^. In conclusion, a higher CXCL16 expression was associated with macrophage- and angiogenesis-related genes and aggressive phenotypes in PTC. Targeting CXCL16 may be a good therapeutic strategy for advanced thyroid cancer.

## Introduction

The tumor microenvironment is comprised of a heterogeneous cell population that includes cancer cells, fibroblasts, endothelial cells, and various immune cells. It has been well established that the bidirectional interactions between cancer cells and their adjacent stromal cells are mainly mediated by various cytokines or growth factors in a paracrine manner, thereby affecting cancer growth and metastasis^1,2^.

Chemokines are chemotactic cytokines that originally are known to have roles in leukocyte recruitment in inflammation-related human pathophysiology^3^. The interaction of chemokines and their receptors recruit leukocytes into tumor microenvironment resulting in the inflammation^2–4^. Among approximately 50 human chemokines, chemokine (C-X-C motif) ligand 16 (CXCL16) is a relatively large chemokine comprised of 254 amino acids and has been identified to bind with the chemokine receptor CXCR6. Unlike other chemokines, CXCL16 is expressed as not only a membrane-bound molecule but also a soluble chemokine^5^. CXCL16 is produced by macrophages and dendritic cells and regulates immune cell chemotaxis into CXCL16-enriched environments^6–9^. Still, the literature regarding the role of CXCL16 in human cancers is conflicting. The expression of CXCL16 and/or CXCR6 has been shown to be positively correlated with poor prognosis-related factors, such as a higher TNM staging or lymph node metastasis in human cancers including prostate, breast, lung, and thyroid cancers^6–8^. However, several studies have reported contradictory findings^10–12^ suggesting that further mechanistic studies are needed to clarify this issue. Recently, several experimental studies demonstrated that CXCL16 was dominantly secreted from stromal cells such as fibroblasts and myeloid cells^13,14^ and supported tumor cell migration and invasion^9,15^.

Thyroid cancer is the most common endocrine cancer with an increasing incidence worldwide^16^. Although most patients with differentiated thyroid cancer have a good clinical prognosis, more than 3–5% of them experience recurrence or metastatic disease^17^. Surgery and radioactive iodine (RAI) treatment are a long-standing treatment of choice for these patients. However, approximately 10% of recurrent or metastatic diseases are refractory to RAI and show a poor prognosis. Currently, tyrosine kinase inhibitors including lenvatinib and sorafenib are available for these patients, but their survival benefits are limited at 11 and 8 months, respectively^18,19^. Therefore, the development of a new treatment target is urgently needed. Indeed, a large body of evidence suggests that several chemokines such as CXCL8 play pro-tumorigenic roles and might be a potent therapeutic target^4^.

The aim of this study was to investigate the molecular characteristics of CXCL16-expressing papillary thyroid cancers (PTCs) and to explore the therapeutic potential of targeting CXCL16 in PTCs. Here, we investigated *CXCL16* expression in the RNA sequencing dataset of PTCs and derived a 3-gene panel for predicting the PTC prognosis. The therapeutic potential of targeting CXCL16 was explored using the murine ectopic tumor model *in vivo*.

## Results

### Enhanced CXCL16 expression in thyroid cancer tissues compared to benign adenomas or normal thyroid tissues

**Figure 1:**
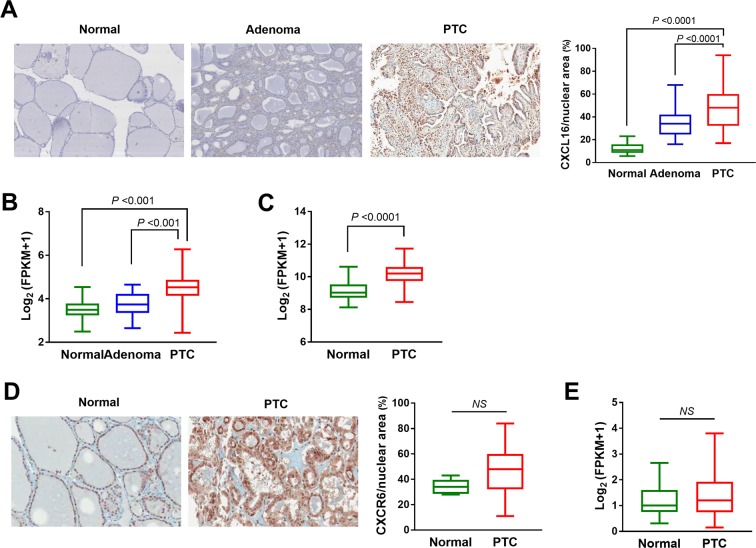
CXCL16 expression in various thyroid tissues: (**A**) Immunohistochemical staining of CXCL16. Representative images and quantification of CXCL16^+^ cells per nuclear area (%) in normal thyroid tissues, benign adenomas, and PTCs. (**B**) mRNA expression of *CXCL16* in normal thyroid tissues, adenomas, and PTCs of SNUH dataset. (**C**) mRNA expression of *CXCL16* in normal thyroid tissues and PTCs of TCGA dataset. (**D**) Immunohistochemical staining of CXCR6 in normal thyroid tissues and PTCs. (**E**) mRNA expression of *CXCR6* in normal thyroid tissues and PTCs of SNUH dataset.

To compare the expressions of CXCL16 in various thyroid tissues, immunohistochemical staining was performed using the anti-CXCL16 antibody on a tissue microarray comprised of 21 normal thyroid tissues, 40 benign adenomas, and 148 PTCs. The protein expressions of CXCL16 were significantly increased in the PTCs compared to that of the benign adenomas (P < 0.001) or normal thyroid tissues (P < 0.001, Fig. 1A). Moreover, analysis of the RNA sequencing data of the SNUH cohort, containing 81 normal thyroid tissues, 25 thyroid adenomas, and 77 PTCs showed that *CXCL16* was significantly upregulated in the PTCs compared to the normal thyroid tissues or benign adenomas (P < 0.001, Fig. 1B). Additionally, a similar finding was observed in The Cancer Genome Atlas (TCGA) dataset which includes 50 normal thyroid tissues and 492 PTCs. *CXCL16* was significantly upregulated in the PTCs compared to the normal thyroid tissues (P < 0.001, Fig. 1C). The expression of CXCR6, a receptor for CXCL16, was previously demonstrated in PTC tissues^9^. It was expressed both cancer and stromal cells in PTC tumor microenvironments. Here, we compared CXCR6 expressions between normal and PTC tissues. Immunohistochemical staining showed that the expression levels were similar between normal thyroid epithelial cells and PTC cancer cells (Fig. 1D). Additionally, *CXCR6* was also similarly expressed in normal thyroid tissues and PTCs in the RNA sequencing data of the SNUH cohort (Fig. 1E).

### Higher mRNA expression of CXCL16 was associated with poor prognostic factors in human PTC

To investigate the clinicopathologic characteristics of CXCL16 expressing PTCs, we further analyzed the TCGA dataset. A total of 492 PTCs were divided into CXCL16^Low^ and CXCL16^High^ groups based on the median *CXCL16* expression value, and then we compared the clinicopathological characteristics between the two groups (Table 1). The CXCL16^High^ group exhibited aggressive pathologic phenotypes, including tall-cell variant, extrathyroidal extension, lymph node metastasis, and higher TNM staging, compared with the CXCL16^Low^ group (P < 0.001; Table 1). The CXCL16^High^ group showed a higher frequency of the *BRAF*^V600E^ mutation compared to the CXCL16^Low^ group (63.3% vs 31.2%, P < 0.001; Table 1). Moreover, *CXCL16* expression showed a significant negative association with the *BRAF*^V600E^-*RAS* score (BRS) (r = −0.772, P < 0.001), indicating that tumors in the CXCL16^High^ group had the *BRAF*^V600E^-like transcriptional profile. Taken together, a higher expression of CXCL16 was associated with poor clinicopathological prognostic factors.

**Table 1:** Clinicopathological characteristics of patients according to mRNA expression level of *CXCL16*: TCGA cohort dataset.

	Total (n = 492)	CXCL16^Low^ (n =246)	CXCL16^High^ (n = 246)	P
Age at diagnosis (yrs)	47.1 ± 15.8	47.4 ± 15.8	46.8 ± 15.9	0.692
Sex, male, n(%)	132 (26.8)	62 (25.2)	70 (28.5)	0.416
**Histological type**, **n**(**%**)				
Classical	349 (70.9)	148 (60.2)	201 (81.7)	<0.001
Follicular variant	99 (20.1)	88 (35.8)	11 (4.5)	
Tall-cell variant	35 (7.1)	5 (2.0)	30 (12.2)	
Other variant	9 (1.8)	5 (2.0)	4 (1.6)	
*BRAF*^V600E^, n(%)	234 (47.4)	77 (31.2)	157 (63.6)	<0.001
Tumor Size (cm)	2.8 ± 1.6	2.8 ± 1.6	2.8 ± 1.7	0.815
Multifocality, n(%)	220/482 (45.6)	118/241 (49.0)	102/241 (42.3)	0.143
Extrathyroidal extension, n(%)	149/476 (31.3)	54/236 (22.9)	95/240 (39.6)	<0.001
Lymph node metastasis	222/445 (49.9)	78/210 (37.1)	144/235 (61.3)	<0.001
Distant metastasis, n(%)	8/265 (3.0)	5/121 (4.1)	3/144 (2.1)	0.475
TNM staging, n(%)				<0.001
I-II	331/490 (67.6)	183/244 (75.0)	148/246 (60.2)	
III-IV	159/490 (32.4)	61/244 (25.0)	98/246 (39.8)	
ATA risk, n(%)				<0.001
Low	171/453 (37.7)	115/227 (50.7)	56/226 (24.8)	
Intermediate	258/453 (57.0)	100/227 (44.1)	158/226 (69.9)	
High	24/453 (5.3)	12/227 (5.3)	12/226 (5.3)	
Recurrence, n(%)	27/454 (5.9)	11/227 (4.8)	16/227 (7.0)	0.321
All-cause mortality, n(%)	14/490 (2.9)	7/244 (2.9)	7/246 (2.8)	0.988

### Higher mRNA expression of CXCL16 was associated with M2 macrophage- and angiogenesis-related genes in human PTC

**Figure 2:**
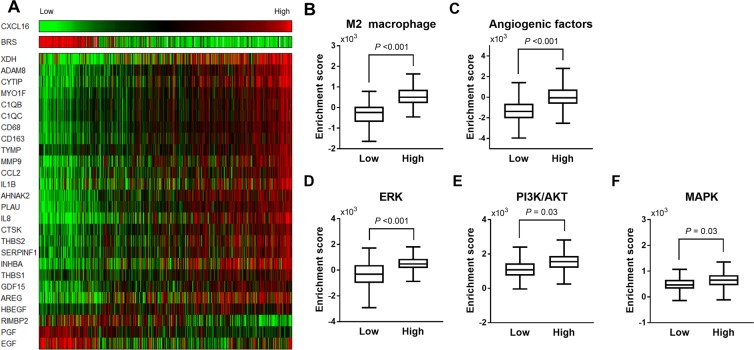
Higher *CXCL16* expressions were associated with M2 macrophage and angiogenesis-related genes and in human PTC tissues of TCGA dataset: (**A**) Heat map analysis of the *BRAF*^V600E^-*RAS* score (BRS), M2 macrophage- and angiogenesis-related gene expressions according to *CXCL16* expressions; (**B**) M2-macrophage; (**C**) Angiogenesis; (**D**) ERK pathway; (**E**) PI3K/AKT pathways; (**F**) MAPK pathways signature in the CXCL16^High^ and CXCL16^Low^ groups were compared using single sample gene set enrichment analysis.

To further explore the molecular characteristics of the CXCL16^High^ group, we then analyzed 120 genes including 55 M2 macrophage-related genes and 65 angiogenesis-related genes^20^, because enhanced infiltration of the M2 macrophages and increased angiogenesis are well-established contributors of tumor aggressiveness in PTCs^21,22^. A total of 120 genes were screened, and 26 genes were differentially expressed between the CXCL16^Low^ and CXCL16^High^ groups (Fig. 2A). Analysis of the differentially expressed genes (DEGs) revealed that 11 M2 macrophage-related genes and 12 angiogenesis-related genes were significantly upregulated, and 1 M2 macrophage-related gene (*RIMBP2*) and 2 angiogenesis-related genes (*PGF* and *EGF*) were significantly downregulated in the CXCL16^High^ group compared with the CXCL16^Low^ group (Fig. 2A and Supplementary Table S1). Furthermore, single sample gene set enrichment analysis (ssGSEA) showed that the M2 macrophage gene signature including 68 genes (Fig. 2B) and the angiogenesis-related gene signature including 65 genes (Fig. 2C) were highly expressed in the CXCL16^High^ group compared with the CXCL16^Low^ group, respectively. Pathway analyses showed that the ERK score (P < 0.001; Fig. 2D), PI3K/AKT (P = 0.03; Fig. 2E) and MAPK (P = 0.03; Fig. 2F) pathway-related genes from the ssGSEA were significantly upregulated in the CXCL16^High^ group compared with the CXCL16^Low^ group.

### Combination of CXCL16 and related genes predicted recurrence-free survival of PTC

**Figure 3:**
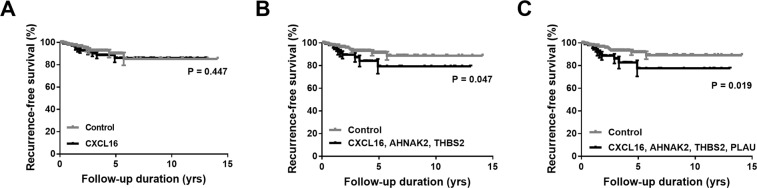
Higher expressions of *CXCL16*, *AHNAK2*, and *THBS2* were associated with poor prognosis in human PTCs from TCGA dataset. Kaplan-Meier curve of recurrence-free survivals for *CXCL16* alone or combination with related genes. (**A**) *CXCL16*; (**B**) *CXCL16*, *AHNAK2*, and *THBS2*; (**C**) *CXCL16*, *AHNAK2*, *THBS2*, and *PLAU*.

Because higher expression of *CXCL16* by itself was not significantly associated with cancer recurrence (Table 1 and Fig. 3A), we then made a predictive gene panel by combining *CXCL16* and its related DEGs. Interestingly, the combination of 3 genes, *CXCL16*, *AHNAK2*, and *THBS2*, showed a significant value of predicting disease recurrence (Fig. 3B). Higher expression of all 3 genes, *CXCL16*, *AHNAK2*, and *THBS2*, showed a shorter recurrence-free survival compared with the others (P = 0.047). Furthermore, adding other fourth genes including *C1QC*, *TYMP*, *PLAU*, *MMP9*, *CYTIP*, or *ADAM8* to the gene panel reinforced its predictive value. Figure 3C shows the representative Kaplan-Meier curve using the 4-gene panel. PTCs with a higher expression of the *CXCL16*, *AHNAK2*, *THBS*, and *PLAU* genes showed a shorter recurrence-free survival than that of the others (P = 0.019).

### Therapeutic effects of blocking CXCL16 in PTC tumors *in vivo*

**Figure 4:**
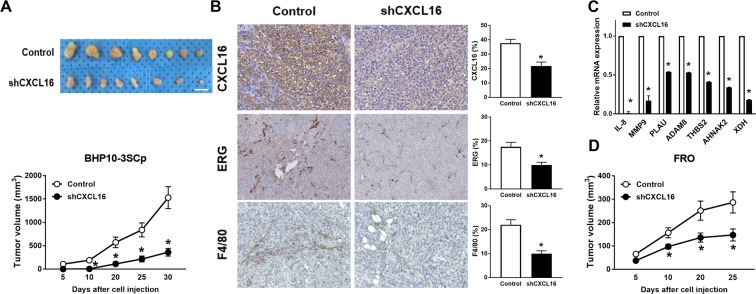
Blocking CXCL16 using shCXCL16 reduced tumor growth of PTCs in a xenograft mouse model. (**A**) BHP10-3SCp^shCXCL16^ or BHP10-3Sp^control^ cells were subcutaneously transplanted in nude mice (n = 7~9 in each group). Representative images and growth curves of tumors from BHP10-3SCp^shCXCL16^ and BHP10-3Sp^control^ cells (scale bar = 1 cm); (**B**) IHC staining with anti-CXCL16, anti-ERG, and anti-F4/80 antibodies in xenografts of (**A**) was performed. Representative images (magnification, x400) and percentage of CXCL16^+^, ERG^+^, F4/80^+^ cells; (**C**) mRNA expression of M2 macrophage and angiogenesis-related genes in xenograft; (**D**) FRO^shCXCL16^ or FRO^control^ cells were subcutaneously transplanted in nude mice (n = 8 in each group). Growth curves of tumors from FRO^shCXCL16^ and FRO^control^ cells. *P < 0.05 compared with control group.

To investigate the therapeutic potential of blocking CXCL16 in advanced thyroid cancer, animal experiments were performed using murine ectopic tumor xenograft models. First, shCXCL16 was transduced into two thyroid cancer cell lines, BHP10-3SCp and FRO, resulting in the genetic depletion of endogenous CXCL16. The ELISA assay showed that the secretory CXCL16 concentrations were decreased in the conditioned medium of the BHP10-3SCp^shCXCL16^ and FRO ^shCXCL16^ cells by 67% and 96% compared to those of BHP10-3SCp^control^ and FRO^control^ cells, respectively (Supplementary Fig. S1A,B). Cell viabilities were significantly decreased by 79.2% in the BHP10-3SCp^shCXCL16^ cells and 57.4% in the FRO ^shCXCL16^ cells at 72 h compared to each of the control cells, respectively (Supplementary Fig. S1C,D). The ectopic tumor model showed that tumors from the BHP10-3SCp ^shCXCL16^ cells exhibited significantly delayed tumor growths from day 10 to day 30 compared to that from the BHP10-3SCp^control^ cells (Fig. 4A). Immunohistochemical staining showed that not only CXCL16^+^ but also ERG^+^ endothelial cells and F4/80^+^ macrophages were significantly reduced in the tumors from the BHP10-3SCp^shCXCL16^ cells compared with those from the BHP10-3SCp^control^ cells (Fig. 4B).

To verify whether the DEGs between the CXCL16^Low^ and CXCL16^High^ groups in the TCGA dataset were directly modulated by CXCL16, total RNAs from the ectopic tumors of the BHP10-3SCp^control^ and BHP10-3SCp^shCXCL16^ cells were harvested. Interestingly, M2-macrophage and/or angiogenesis-related DEGs such as *IL-8*, *MMP9*, *PLAU*, *ADAM±8*, *THBS2*, *AHNAK2*, and *XDH* were significantly downregulated in the tumors of the BHP10-3SCp^shCXCL16^ cells compared with those from the BHP10-3SCp^control^ cells (Fig. 4C). Consistently, tumor growth was significantly delayed in the tumors from the FRO^shCXCL16^ cells compared with those from the FRO^control^ cells (Fig. 4D).

Previous study showed that both human PTC and monocyte THP-1 cells expressing *CXCR6*, and treatment of conditioned medium of co-cultures of monocyte and BHP10-3SCp cells up-regulated *CXCR6* expressions in both cells^9^. Thus, we compared the expression level of *CXCR6* and demonstrated that the expression was significantly higher in THP-1 cells than those in normal thyroid epithelial or thyroid cancer cell lines including BHP10-3SCp and FRO cells (Supplementary Fig. S2). Furthermore, CXCL16 enhanced cell migration potentials of THP-1 cells without change of cell viability (Supplementary Fig. S3), suggesting that PTC-producing CXCL16 may recruit macrophages into PTC microenvironments.

**Figure 5:**
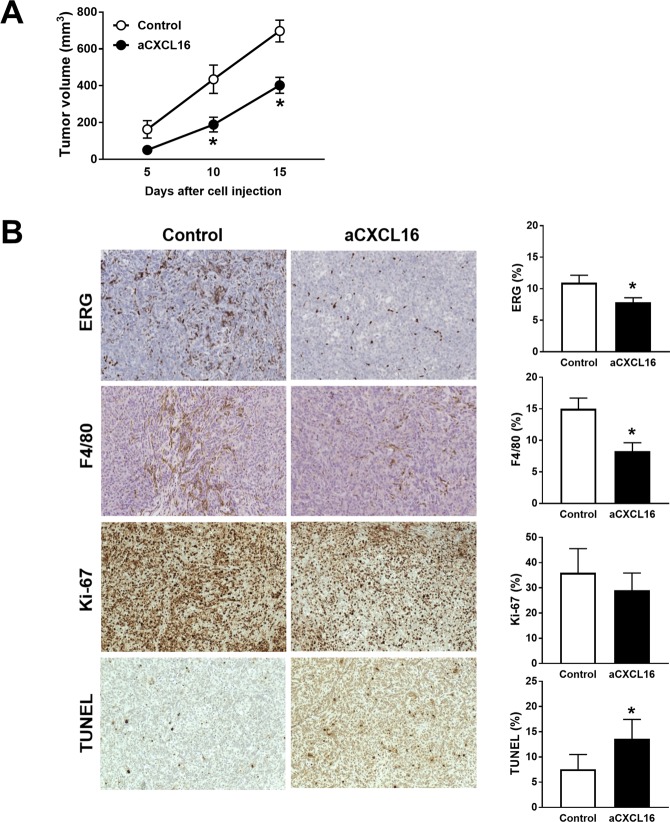
Blocking CXCL16 using anti-CXCL16 antibody (aCXCL16) reduced tumor growth of PTCs in a xenograft mouse model. BHP10-3SCp cells were co-transplanted with macrophages in nude mice, and aCXCL16 or anti-IgG (control) were injected intraperitoneally. (**A**) Comparing tumor growth between aCXCL16 and control groups; (**B**) Immunohistochemical staining for ERG, F4/80, and Ki-67 and TUNEL assay were performed in xenograft tumors. Representative images (magnification, x400) and percentage of ERG^+^, F4/80^+^, Ki-67^+^, and TUNEL^+^ cells. *P < 0.05 compared with control group.

Because CXCL16 is produced not only from cancer cells but also from various stromal cells including macrophages or fibroblasts^9,23^, we then depleted it by using anti-CXCL16 neutralizing antibody in a macrophage-laden xenograft tumor model. Tumorigenic clones of BHP10-3SCp cells were transplanted with human monocytes, and anti-CXCL16 antibody (aCXCL16) was injected once per week. The tumor volumes were significantly reduced at day 10 (435 ± 154 mm^3^ vs 234 ± 44 mm^3^, P = 0.03) and day 15 (697 ± 118 mm^3^ vs 402 ± 97 mm^3^, P < 0.01; Fig. 5A). Consistently, ERG^+^ endothelial cells and F4/80^+^ macrophages were significantly decreased in the aCXCL16-treated group compared with the control group (P < 0.05; Fig. 5B). Moreover, TUNEL staining showed that cell apoptosis was significantly increased (P < 0.05; Fig. 5B), and proliferating Ki-67 positive cells were decreased with marginal significance (P = 0.07; Fig. 5B) in aCXCL16-treated group compared with the control group. Collectively, the inhibitions of CXCL16 by targeting either the tumor cells or tumor microenvironment could be an effective therapeutic strategy for advanced thyroid cancer.

## Discussion

Cumulative evidence has demonstrated that various cytokines mediate pro-tumorigenic actions in a tissue-specific manner. In the present study, analyses of the TCGA dataset demonstrated that a higher *CXCL16* expression was positively correlated with M2 macrophage- and angiogenesis-related gene expressions and associated with poor prognostic factors including the *BRAF*^V600E^ mutation and a higher TNM staging. Although CXCL16 alone did not predict the disease prognosis, a combination of 3 genes including *CXCL16* and 2 related DEGs, *AHNAK2*, and *THBS2*, predicted recurrence-free survivals. In murine ectopic tumor models, CXCL16-depleted PTC cells exhibited a significantly reduced tumor growth with decreased macrophage infiltration and tumor angiogenesis. Moreover, upregulated CXCL16-related DEGs from the human study tumors were significantly decreased in the tumors from the CXCL16-depleted cells, indicating that CXCL16 may actively modulate the protumorigenic conditioning of the PTC tumor microenvironments. Finally, treatment of anti-CXCL16 blocking antibodies significantly inhibited the tumor growth of macrophage-laden PTCs in the murine model. Taken together, a 3-gene panel that includes *CXCL16* can predict a poor prognosis for PTCs, and targeting CXCL16 may be a good therapeutic strategy for advanced thyroid cancers.

CXCL16 is a recently characterized pro-angiogenic cytokine in various human pathophysiologies. CXCL16 induces endothelial cell proliferation, migration, and new vessel formation *in vitro*^24,25^. In a murine rheumatoid arthritis model, CXCL16/CXCR6 signaling has been reported to have a central role in endothelial progenitor cell chemotaxis and angiogenesis^26^. CXCL16 also acts as an important angiogenic factor in the tumor microenvironment. Tumor cell-conditioned myeloid suppressor cells were shown to produce CCL2 and CXCL16, which enhanced angiogenesis^27^. In a hepatocellular carcinoma xenograft mouse model, suppression of CXCR6, a receptor for CXCL16, reduced tumor angiogenesis^28^. In the present study, we first reported that targeting CXCL16 either in cancer cells using shRNA or in the microenvironment using a neutralizing antibody efficiently blocked tumor growth and angiogenesis in thyroid cancers. Tumor angiogenesis has an important role in the progression of thyroid cancers^21^. Multi-targeted tyrosine kinase inhibitors including lenvatinib and sorafenib, currently used in RAI-refractory advanced thyroid cancer, are anti-angiogenic agents that mainly target VEGF receptor. Therefore, CXCL16 can be another target for anti-angiogenic therapy in advanced thyroid cancer, and patients with higher CXCL16 expression might be selected for challenge targeting CXCL16 as a precision medicine approach.

A previous study demonstrated CXCL16 as a potential mediator of tumor associated macrophages (TAMs), supporting TAM-mediated cell migration and the invasion potentials of PTCs^9^. The present study verified that the expression of *CXCL16* was positively associated with TAM-related and *BRAF*^V600E^-like signature genes in human PTCs. Because TAMs have been established to have pro-tumorigenic roles in various human cancers including thyroid cancer^29^, the targeting of TAMs as an anti-cancer therapy has been investigated intensively at both the cellular and molecular levels^30^. The first approach was targeting macrophages themselves by systemic depletion. However, this prolonged systemic depletion of macrophages may result in host immune suppression and sensitization to opportunistic infections^30^. Thus, targeting the downstream mediators of TAMs may be a good alternative strategy. Several angiogenic factors including VEGF-A and MMP9 mediate the actions of the TAMs^29^, depending on the tissue or organ. Therefore, tissue-specific TAM mediators need to be identified and validated in each specific organ. Fitting the latter approach of targeting TAMs, CXCL16 might be a good cancer treatment target for advanced thyroid cancer. One of the important finding in this study was that CXCL16-related DEGs from the TCGA dataset which are M2-macrophage and/or angiogenesis related genes were down-regulated in xenograft tumors from CXCL16-depleting cells. It suggested that CXCL16 is the essential player in TAM-enriched tumor microenvironment and can be a potent therapeutic target.

One of the strengths of this study is that it showed that the clinical implication of *CXCL16* expression was validated as a prognostic marker for human PTCs. Indeed, tumor microenvironments are a very heterogeneous complex, comprised of various cell types which secretes numerous cytokines and growth factors^1,31^. Therefore, predicting a value for a single factor might have a limitation. The present study demonstrated an acceptable predicting value of a genetic panel for PTC prognosis comprised of 2 related genes to CXCL16. Further studies are need to apply this genetic panel to human PTCs.

In conclusion, a higher CXCL16 expression was associated with macrophage-, angiogenesis-, and *BRAF*^V600E^-like signature genes in human PTCs. A three-gene panel related to CXCL16 can be used to predict the disease prognosis, and targeting CXCL16 may be a good therapeutic strategy for macrophage-enriched, advanced thyroid cancer.

## Methods

### Thyroid tissue microarrays

Thyroid tissue microarrays that had been constructed as previously described^32^ were used in this study. Briefly, blocks of paraffin-embedded thyroid tissues were obtained from patients who underwent thyroid surgery from January 1993 to December 2003 at Seoul National University Boramae Medical Center and Seoul National University Hospital.

### Processing of genomic data from the SNUH and TCGA datasets

We used mRNA expression data from 77 PTCs, 25 thyroid follicular adenomas, and 81 normal thyroid tissues from the SNUH cohort. The process of RNA sequencing has been described in detail previously^33^. As a validation set, we used the mRNA expression data from the TCGA; clinical information and mRNA expression data obtained by RNA sequencing from 492 PTCs and 59 paired normal thyroid tissues were downloaded from the UCSC Cancer Browser (https://genome-cancer.ucsc.edu) on October 12, 2017. DEGs were defined by DESeq2 to have a q-value < 0.05, |Log_2_(fold change)| ≥ 1, and a baseMean ≥ 100, using normalized raw counts of sequencing reads by the regularized log transformation method^34^. To adjust for the multiple testing, the q-value calculated by Benjamini–Hochberg correction was used. To construct a heatmap, the centered rlog values were applied to hierarchical clustering using Cluster 3.0^35^. To quantify the degree of similarity of the gene expression profile of a given tumor to the *BRAF*^V600E^ or *RAS* mutation profile, BRS, an mRNA-based signature score that was developed by the TCGA study^36^, was used. Tumors with a negative BRS were defined as *BRAF*^V600E^-like while tumors with a positive BRS were defined as *RAS*-like.

In addition, ssGSEA from GenePattern (http://software.broadinstitute.org/cancer/software/genepattern/) was implemented to evaluate the activation of several signaling pathways for each sample^37^. Before the analysis, we excluded genes with a low expression level across whole samples according to the baseMean value (<100) from DESeq2^34^. For the PI3K/AKT and MAPK pathways, we used the Kyoto Encyclopedia of Genes and Genomes (KEGG) database which was provided by The Molecular Signatures Database (MSigDB)^38,39^. For M2 macrophage and angiogenesis, we used custom gene lists based on previous studies^20,40,41^. The ERK score was also calculated by ssGSEA with 52 gene lists from the TCGA study^36,42^.

### Progression prediction models

To predict the recurrence with the expression of *CXCL16*, M2 macrophage-related genes, and angiogenesis-related genes, each gene expression was divided into two groups (high and low group) based on the median expression value of each gene, and combinations from 2 to 4 genes were made. All combinations of genes were examined to find a significant association with the recurrence. Recurrence-free survival of PTCs with a higher expression of genes was compared to the others using Kaplan-Meier survival analysis.

### Immunohistochemical staining

Formalin-fixed, paraffin-embedded tissue sections from tissue microarrays or xenograft tumors were stained using immunohistochemistry for CXCL16 (Abcam, Cambridge, MA, USA; dilution ratio 1:100), ERG (Ventana Medical Systems, Tucson, AZ, USA), F4/80 (eBioscience, San Diego, CA, USA, dilution ratio 1:200), and Ki-67 (Neomarkers, Fremont CA, USA, dilution ratio 1:500), using the BenchMark XT automated immunohistochemistry slide staining system (Ventana) according to the manufacturer’s instructions. TUNEL assay (Millipore, MA, USA) was used to detect the apoptotic cells in xenograft tumor sections. To analyze the immunoreactivities, the core tumor areas were divided into quarters, and 5 areas were randomly chosen from each quarter and the central area. Under 400x magnification, immunoreactive cells were counted by two different medical doctors including one pathologist and expressed as the percentage of positive cells per area.

### Animal studies

Six-week-old female BALB/c nude mice (Orient Bio, Seongnam-si, Korea) were purchased and used in this study. To establish thyroid cancer xenograft models, cells (5 × 10^6^/100 μL PBS) or FRO cells (2 × 10^6^/100 μL PBS) harboring shCXCL16 or the control, were mixed with growth factor-reduced Matrigel (70 μL, 4°C; BD Biosciences, San Jose CA, USA) and transplanted into the dorsal skin fold in nude mice. For macrophage-laden ectopic tumors, THP-1 cells (1.25 × 10^6^/100 μL PBS) were co-transplanted with BHP10-3SCp cells. Anti-CXCL16 antibody or IgG (R&D Systems, Minneapolis, MN, USA) was injected intraperitoneally once a week after the tumor cells were transplanted. We measured the tumor size with a caliper and calculated the tumor volume with using the following equation: volume = ½ × *a* × *b*^2^, where *a* = long tumor diameter and *b* = short tumor diameter^43^. After 5 to 6 weeks, the mice were euthanized, and the tumors were surgically removed and fixed with 10% formalin.

### Cell cultures

The human PTC cell line, BHP10-3SCp, which is a tumorigenic clone of the BHP10-3M cell line containing the *RET/PTC* rearrangement, were developed and kindly provided by Dr. Soon-Hyun Ahn (Seoul National University College of Medicine, Seoul, Korea) and Dr. Gary L. Clayman (MD Anderson Cancer Center, Houston, TX, USA)^44^. FRO cells (anaplastic thyroid cancer cell) harboring *BRAF*^V600E^ and *TERT*^C250T^ mutation and THP-1 cells (human monocyte/macrophages) harboring *CDKN2A*, *RAS*, and *TP53* mutation were kind gifts from Dr. June-Key Chung and Dr. Hyo-Soo Kim (Seoul National University College of Medicine, Seoul, Republic of Korea), respectively. All cells were cultured in Roswell Park Memorial Institute (RPMI)-1640 medium supplemented with 10% fetal bovine serum (FBS).

### Transduction of CXCL16 shRNA

To deplete the endogenous expression of CXCL16 in thyroid cancer cells, CXCL16 shRNA was stably transduced into BHP10-3SCp and FRO cells. Briefly, human CXCL16–specific shRNA (Mission TRCN0000057990 and TRCN0000057991, 1 × 10^6^ TU/mL, pLKO.1 vector) and non-target shRNA control (SHC003) lentiviral transduction particles were obtained from Sigma-Aldrich (St. Louis, MO, USA). Cells were plated on 96-well plates at a concentration of 1.6 × 10^4^ cells/well and infected with lentiviral particles (MOI 5) expressing the specific shRNA. After 2 days, the cells were selected with puromycin (Sigma-Aldrich) until complete death of the uninfected cells was observed.

### RNA extraction and RT-PCR analysis

Trizol (Invitrogen, Carlsbad, CA, USA) was used to harvest mRNA from the xenograft tumors and cells. RT-PCR was performed using a Perkin-Elmer GeneAmp PCR System 9600 (Waltham, MA, USA). The PCR primer sets are listed in Supplementary Table S2.

### Statistical analysis

Data are presented as the means and standard deviations. We analyzed the continuous variables by the Kruskal–Wallis test or the Mann–Whitney U test. Recurrence-free survival curves were plotted using the Kaplan-Meier method and compared using the log-rank test. The statistical analysis was performed using SPSS version 22.0 software for Windows (SPSS, Chicago, IL, USA), and a *P* < 0.05 was considered statistically significant.

### Ethic statement

The experiments with human thyroid tissues were approved by the institutional review boards of Seoul National University Boramae Medical Center (06-2010-176) and Seoul National University Hospital (1107-060-369). Informed consent was obtained from all patients after explanation of the purpose and procedure of the study. The animal experimental protocols were approved by the Institutional Animal Care and Use Committee of Seoul National University (SNU-160203-1). All methods involving humans and animals in this study were performed in accordance with relevant guidelines and regulations.

## Supplementary information

Supplementary Figure S1, Figure S2, Figure S3, Table S1, and Table S2
