# Supplementary material for: CXCL16 positively correlated with M2-macrophage infiltration, enhanced angiogenesis, and poor prognosis in thyroid cancer
Source: Sci Rep. 2019 Sep 16;9:13288. doi: 10.1038/s41598-019-49613-z (PMC6746802; doi:10.1038/s41598-019-49613-z)
Supplement: Supplementary file 1 — Supplementary Figure S1, Figure S2, Figure S3, Table S1, and Table S2 [file 41598_2019_49613_MOESM1_ESM.doc]

**CXCL16 positively correlated with M2-macrophage infiltration, enhanced angiogenesis, and poor prognosis in thyroid cancer**

Min Joo Kim^1^, Hyun Jin Sun^1^, Young Shin Song^1, 2^, Seong-Keun Yoo^3^, Young A Kim^4^, [Jeong-Sun Seo](https://www.ncbi.nlm.nih.gov/pubmed/?term=Seo JS%5BAuthor%5D&cauthor=true&cauthor_uid=27494611)^3^, Young Joo Park^1,5^, Sun Wook Cho^1, *^

^1^Department of Internal Medicine, Seoul National University Hospital, 101, Daehak-ro, Jongno-gu, Seoul 03080, Republic of Korea

^2^Department of Internal Medicine, CHA Bundang Medical Center, CHA University, 59, Yatap-ro, Bundang-gu, Seongnam, Republic of Korea

^3^Gong Wu Genomic Medicine Institute, Seoul National University Bundang Hospital, Dolma-ro 172, Seongnam 13605, Republic of Korea

^4^Department of Pathology, Boramae Medical Center, 20, Boramae-ro 5-gil, Dongjak-gu, Seoul 07061, Republic of Korea

^5^1Genomic Medicine Institute, Medical Research Center, Seoul National University, Seoul, Republic of Korea

555 Department of Internal Medicine, Seoul National University College of Medicine, 101, Daehak-ro, Jongno-gu, Seoul 03080, Republic of Korea

*Corresponding author. [swchomd@snu.ac.kr](mailto:swchomd@snu.ac.kr)

**Method for supplementary figure**

**Measurement of CXCL16**

CXCL16 concentration for conditioned medium of BHP10-3SCp and FRO cells was measured using an ELISA kit (R&D Systems Inc.,Minneapolis, MN, USA).

**Cell viability and migration assay**

Cells were seeded onto a 96-well tissue culture plate. After 48 h, cell viability was evaluated using a CCK-8 assay (Dojindo, Kumamoto, Japan). To evaluate the migration potential of THP-1 cells, transwell migration was performed using 8 mm pore-size polycarbonate membrane (Corning, NY, USA) as previously described.

**Cells for RNA study**

Trizol (Invitrogen, Carlsbad, CA, USA) was used to harvest mRNA from cells. RT-PCR was performed using a Perkin-Elmer GeneAmp PCR System 9600 (Waltham, MA, USA). The PCR primer for *CXCR6* was 5′-ATG CCA TGA CCA GCT TTC ACT -3′ (forward) and 5′- TTA AGG CAG GCC CTC AGG TA -3′ (reverse).

**
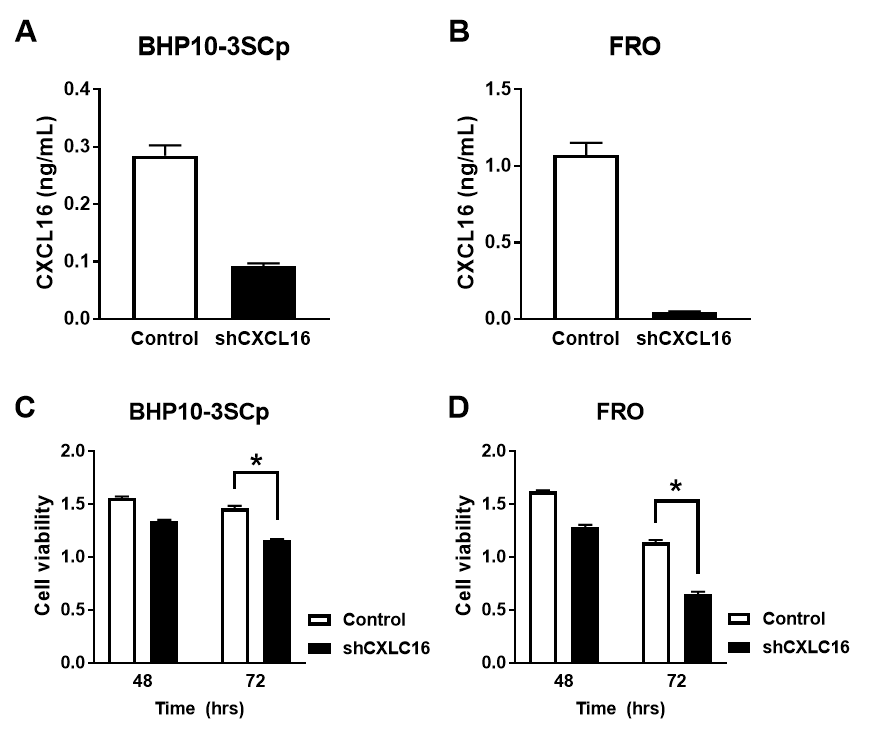
**

**Supplementary Figure S1.** Blocking endogenous CXCL16 using shCXCL16 reduced cell viability. (**A−B**) CXCL16 concentrations from conditioned medium of (**A**) BHP10-3SCp^shCXCL16^ and BHP10-3Sp^control^ cells; (**B**) FRO^shCXCL16^ and FRO^control^ cells. (**C−D**) Cell viabilities were compared at 48, 72 hrs between (**C**) BHP10-3SCp^shCXCL16^ and BHP10-3Sp^control^ cells; (**D**) FRO^shCXCL16^ and FRO^control^ cells.


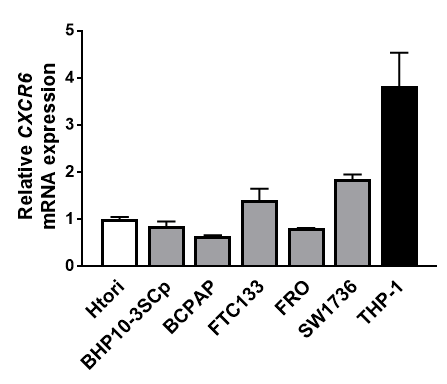


**Supplementary Figure S2.** *CXCR6* expressions in cell-lines of normal thyroid epithelial cell, thyroid cancers, and monocyte.


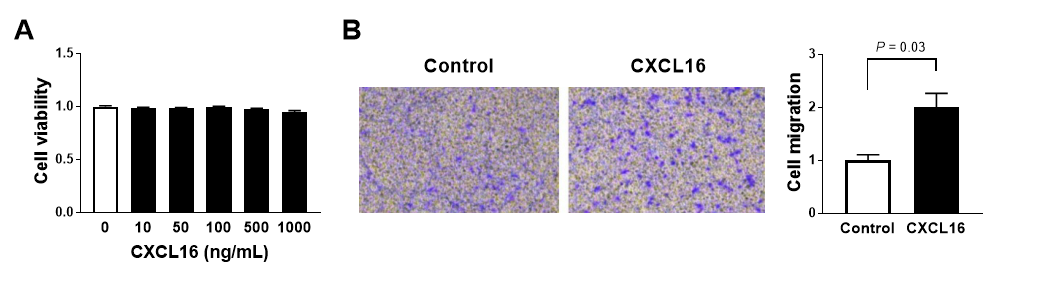


**Supplementary Figure S3.** CXCL16 enhances cell migration potentials of human monocyte/macrophage cell-line. (**A**) Cell viability of THP-1 cells was not change by recombinant human CXCL16 treatment. (**B**) Cell migration of THP-1 cells were enhanced by CXCL16 treatment.

**Supplementary Table S1.** DEGs between CXCL16^Low^ and CXCL16^High^ groups: TCGA cohort dataset

|  | baseMean | Fold change (log2) | SE of log2 fold change | | *p*-value | *p-*adj |
| --- | --- | --- | --- | --- | --- | --- |
| ***CXCL16*** | 3367.9 | 1.07 | 0.04 | | 4.9229E-149 | 9.9212E-145 |
| **M2 macrophage-related genes** | | | |  |  |  |
| *ADAM8* | 421.3 | 1.53 | 0.09 | | 2.35457E-64 | 6.87705E-62 |
| *MYO1F* | 787.8 | 1.31 | 0.09 | | 1.81998E-49 | 1.23912E-47 |
| *AHNAK2* | 6327.5 | 1.82 | 0.13 | | 2.09689E-45 | 1.0257E-43 |
| *C1QB* | 4223.1 | 1.47 | 0.11 | | 1.19167E-43 | 4.90119E-42 |
| *CTSK* | 1686.3 | 1.45 | 0.10 | | 1.76627E-43 | 7.14771E-42 |
| *C1QC* | 3234.7 | 1.35 | 0.10 | | 3.15713E-41 | 1.04134E-39 |
| *CYTIP* | 464.6 | 1.54 | 0.12 | | 1.33812E-37 | 3.42657E-36 |
| *XDH* | 167.8 | 1.79 | 0.21 | | 4.09276E-18 | 2.527E-17 |
| *GDF15* | 6349.5 | 1.07 | 0.13 | | 6.87204E-17 | 3.88368E-16 |
| *CD68* | 3755.0 | 1.46 | 0.09 | | 6.65452E-57 | 8.82293E-55 |
| *CD163* | 1500.1 | 1.24 | 0.10 | | 1.39156E-32 | 2.43861E-31 |
| *RIMBP2* | 217.3 | -1.19 | 0.12 | | 4.84637E-23 | 4.20987E-22 |
|  | | | |  |  |  |
| **Angiogenesis-related genes** | | | |  |  |  |
| *IL8* | 496.8 | 1.94 | 0.14 | | 3.53832E-43 | 1.38731E-41 |
| *THBS2* | 2601.0 | 1.79 | 0.13 | | 6.31159E-42 | 2.17804E-40 |
| *TYMP* | 2210.1 | 1.13 | 0.09 | | 9.34866E-34 | 1.77238E-32 |
| *INHBA* | 203.5 | 1.64 | 0.14 | | 1.93868E-32 | 3.36522E-31 |
| *SERPINF1* | 2210.7 | 1.35 | 0.11 | | 3.36127E-32 | 5.71162E-31 |
| *PLAU* | 10500.5 | 1.50 | 0.13 | | 1.77974E-31 | 2.86021E-30 |
| *THBS1* | 5572.3 | 1.32 | 0.12 | | 3.13788E-30 | 4.59243E-29 |
| *CCL2* | 849.6 | 1.10 | 0.12 | | 1.2055E-21 | 9.62918E-21 |
| *MMP9* | 2366.6 | 1.37 | 0.14 | | 2.16025E-21 | 1.69663E-20 |
| *AREG* | 200.2 | 1.49 | 0.16 | | 6.94046E-21 | 5.28014E-20 |
| *IL1B* | 166.7 | 1.00 | 0.12 | | 7.57222E-16 | 3.97094E-15 |
| *HBEGF* | 3440.9 | 1.01 | 0.13 | | 2.43054E-15 | 1.22212E-14 |
| *PGF* | 4565.0 | -1.48 | 0.12 | | 2.58213E-37 | 6.48849E-36 |
| *EGF* | 114.7 | -1.29 | 0.14 | | 6.22895E-21 | 4.7514E-20 |

**Supplementary Table S2.** Sequences of primers used to amplify each of the genes in RT-PCR

| **Gene** |  | **Primer Sequence (5'→3')** |
| --- | --- | --- |
| *IL-8* | Forward  Reverse | TGT GAA GGT GCA GTT TTG CCA AGG  GTT GGC GCA GTG TGG TCC ACT C |
| *MMP9* | Forward  Reverse | TCG AAC TTT GAC AGC GAC AAG  GCA CTG AGG AAT GAT CTA AGC |
| *PLAU* | Forward  Reverse | CTC ATC CTA CAC AAG GAC TAC  CAG GCA GAT GGT CTG TAT AGT |
| *ADAM8* | Forward  Reverse | CGA TGA TGC TGC CTG CGA TTG  CGC AGG TGG AGG GTG AAG TT |
| *THBS2* | Forward  Reverse | TTA CCG CTT CGT GCG CTT TGA C  AAC AGC GTG CCC CTG GAC TTG |
| *AHNAK2* | Forward  Reverse | TCC TGG TGG AAG CGA GAT TCA G  ACC ACC TGT GAC ACT GTA GCC A |
| *XDH* | Forward  Reverse | GTG GAT GCT GTG GAG GAG AT  TGC TTC CGA GGA GTG TCT TT |
| *GAPDH* | Forward  Reverse | TGC ACC ACC AAC TGC TTA GC  GGC ATG GAC TGT GGT CAT GAG |
